# Sequence Analysis of Six Candidate Genes in Miniature Schnauzers with Primary Hypertriglyceridemia

**DOI:** 10.3390/genes15020193

**Published:** 2024-01-31

**Authors:** Nicole M. Tate, Michaela Underwood, Alison Thomas-Hollands, Katie M. Minor, Jonah N. Cullen, Steven G. Friedenberg, James R. Mickelson, Panagiotis G. Xenoulis, Joerg M. Steiner, Eva Furrow

**Affiliations:** 1 Department of Veterinary Clinical Sciences, College of Veterinary Medicine, University of Minnesota, St. Paul, MN 55108, USA; minork@umn.edu (K.M.M.); fried255@umn.edu (S.G.F.); furro004@umn.edu (E.F.); 2 VCA Veterinary Specialty & Emergency Center of Kalamazoo, Kalamazoo, MI 49001, USA; kaylaunderwood247@gmail.com; 3 VCA CARE Centre, Calgary, AB T2H 2Y4, Canada; 4 Department of Veterinary Population Medicine, College of Veterinary Medicine, University of Minnesota, St. Paul, MN 55108, USA; cull0084@umn.edu; 5 Department of Veterinary and Biomedical Sciences, College of Veterinary Medicine, University of Minnesota, St. Paul, MN 55108, USA; micke001@umn.edu; 6 Clinic of Medicine, Faculty of Veterinary Medicine, University of Thessaly, 43100 Karditsa, Greece; pxenoulis@vet.uth.gr; 7 Gastrointestinal Laboratory, Department of Small Animal Clinical Sciences, School of Veterinary and Biomedical Sciences, Texas A&M University, College Station, TX 77843, USA; jsteiner@cvm.tamu.edu

**Keywords:** hypertriglyceridemia, canine, Miniature Schnauzer, candidate genes

## Abstract

Miniature Schnauzers are predisposed to primary hypertriglyceridemia (HTG). In this study, we performed whole genome sequencing (WGS) of eight Miniature Schnauzers with primary HTG and screened for risk variants in six HTG candidate genes: *LPL*, *APOC2*, *APOA5*, *GPIHBP1*, *LMF1*, and *APOE*. Variants were filtered to identify those present in ≥2 Miniature Schnauzers with primary HTG and uncommon (<10% allele frequency) in a WGS variant database including 613 dogs from 61 other breeds. Three variants passed filtering: an *APOE* TATA box deletion, an *LMF1* intronic SNP, and a *GPIHBP1* missense variant. The *APOE* and *GPIHBP1* variants were genotyped in a cohort of 108 Miniature Schnauzers, including 68 with primary HTG and 40 controls. A multivariable regression model, including age and sex, did not identify an effect of *APOE* (estimate = 0.18, std. error = 0.14; *p* = 0.20) or *GPIHBP1* genotypes (estimate = −0.26, std. error = 0.42; *p* = 0.54) on triglyceride concentration. In conclusion, we did not identify a monogenic cause for primary HTG in Miniature Schnauzers in the six genes evaluated. However, if HTG in Miniature Schnauzers is a complex disease resulting from the cumulative effects of multiple variants and environment, the identified variants cannot be ruled out as contributing factors.

## 1. Introduction

Miniature Schnauzers have a predisposition for primary hypertriglyceridemia (HTG), defined as an elevated triglyceride concentration in the absence of an identifiable underlying cause [1]. In the United States, HTG is common in Miniature Schnauzers, with more than 40% affected by 6 years of age [1]. Complications associated with HTG include pancreatitis, gallbladder mucocele, and glomerular proteinuria [2,3,4]. An underlying genetic risk factor is suspected to be responsible for HTG in this breed. If HTG risk variants are identified, screening would allow for early identification of susceptible dogs and could inform clinical care to prevent the development of HTG and its complications. Genotyping for high-risk variants might also be applied in breeding decisions to reduce the prevalence of HTG in Miniature Schnauzers.

In humans, >50% of patients with severe primary HTG have rare variants in one of five major lipid metabolism genes: lipoprotein lipase (*LPL*), apolipoprotein C-II (*APOC2*), apolipoprotein A-V (*APOA5*), glycosylphosphatidylinositol-anchored HDL-binding protein 1 (*GPIHBP1*), and lipase maturation factor 1 (*LMF1*) [5]. Previous sequencing of *LPL* and *APOC2* did not reveal any variants in Miniature Schnauzers with primary HTG [6,7]. However, the dog genome was not well annotated at the time of the *LPL* sequencing, and not all exons were captured [6]. Also, non-coding regions were not thoroughly evaluated for either gene [6,7]. Non-coding variants, such as core promoter regions, can influence gene expression [8]. Therefore, re-evaluation of *LPL* and *APOC2* and analysis of additional major lipid metabolism genes is warranted in Miniature Schnauzers with primary HTG. Along with the five major susceptibility genes for HTG in humans, apolipoprotein E (*APOE*) is another potential candidate gene for HTG in Miniature Schnauzers. Rare variants in *APOE* cause HTG and lipoprotein glomerulopathy in humans, and similar glomerular lesions occur in Miniature Schnauzers with HTG [9,10].

Whole genome sequencing (WGS) offers a rapid and comprehensive alternative to conventional sequencing of multiple candidate genes [11,12]. The objective of this study was to use WGS to discover putative risk variants in the aforementioned six candidate genes for HTG in Miniature Schnauzers. We hypothesized that we would identify a putative causal variant associated with HTG in Miniature Schnauzer dogs.

## 2. Materials and Methods

### 2.1. Miniature Schnauzers WGS Cohort

Eight Miniature Schnauzers with primary HTG were selected for WGS. This sample size was chosen to capture a greater than 80% probability of detecting a variant present in at least 20% of the population based on basic probability of events:

(1 − (0.8)^n^) (1)

This calculation underestimates the true probability of detecting a variant at this population frequency, as dogs are diploid. The primary HTG cases selected for WGS were dogs with fasting serum triglyceride concentration > 250 mg/dL and no clinical suspicion or previous diagnosis of a condition that can cause HTG (e.g., hypothyroidism, diabetes mellitus, hyperadrenocorticism). All primary HTG dogs were managed by veterinary board-certified internists and had blood samples submitted to the University of Minnesota Canine Genetics Laboratory for hyperlipidemia research. Informed owner consent was obtained, and the University of Minnesota Institutional Animal Care and Use Committee approved the study (Protocol #1509-33019A). Four of these dogs were previously included in an analysis of *APOC2* for coding variants [7], and three dogs were recruited after diagnosis with glomerular lipid thromboemboli [9].

### 2.2. DNA Isolation and WGS

Ethylenediaminetetraacetic acid (EDTA) blood samples (2–4 mL) were obtained for genomic DNA isolation. Genomic DNA was isolated using a commercial kit (Gentra Puregene Blood Kit, Qiagen Sciences, Germantown, MD, USA). WGS was performed using 150 base pair (bp) paired-end reads on an Illumina HiSeq 2500 with an average coverage of 18×. Quality control, mapping, and variant calling were performed using a previously described standardized pipeline [13] and the UU_Cfam_GSD_1.0/canFam4.0 dog reference assembly (GenBank accession GCA_011100685.1) [14]. Variants were annotated using Ensembl’s Variant Effect Predictor (VEP; RRID SCR_007931) [15].

### 2.3. Candidate Gene Analysis

The names and functions of the six candidate genes analyzed in this study are summarized in Table 1. Variants present in two or more cases and located within or up to 150 bp upstream of the candidate genes were extracted [16,17]. Variants were filtered to identify those with a <0.10 allele frequency in non-Miniature Schnauzer breeds; this allele frequency cutoff was chosen on the basis of the low prevalence of HTG in the general dog population [1,18]. This filtering step was accomplished using a private WGS database containing variant calls from 613 dogs of 61 non-Miniature Schnauzer breeds (Table S1). The private WGS database also included 30 Miniature Schnauzers with unknown HTG phenotypes. Variant allele frequencies were calculated separately for the 8 Miniature Schnauzers with primary HTG, the 30 Miniature Schnauzers with unknown phenotypes, and the 613 dogs from 61 non-Miniature Schnauzer breeds.

Variant locations were assessed for base-wise conservation using the “100 Vertebrates Basewise Conservation by phyloP (phyloP100way)” track on the UCSC Genome browser [24]. The absolute values of the scores from phyloP100way are the −log10 (*p*-value) for rejecting the null hypothesis of neutral evolution. Conservation is indicated by positive scores, while negative scores indicate acceleration. The “Vertebrate Multiz Alignment & Conservation (100 Species)” track was used to determine the number of species the base position was conserved across [25]. Two variant pathogenicity prediction methods, SNPs&GO (RRID SCR_005788) and PolyPhen-2 (RRID SCR_013189), were used to assess the pathogenicity of missense variants [26,27]. For both programs, scores >0.5 are considered pathogenic predictions. InterProScan (RRID SCR_005829) was used to determine if variants resided in protein domains or other important sites [28].

### 2.4. Variant Follow-Up Genotyping

Two variants of interest were identified, one in *APOE* and one in *GPIHBP1*. Both were genotyped in a follow-up cohort to determine whether either was associated with primary HTG in the Miniature Schnauzer breed. Samples for the genotyping cohort were selected from Miniature Schnauzers from past and ongoing research projects with DNA biobanked (−80 °C) at the University of Minnesota Canine Genetics Laboratory [4,9,29,30]. Samples were genotyped for the two variants if they had a fasting triglyceride concentration available. Dogs were classified as having HTG if the triglyceride concentration was >108 mg/dL at any age and as a control if the concentration was ≤108 mg/dL at 6 years of age or older [1]. These classifications were only used to describe the study population, as statistical analysis (see below) analyzed triglyceride concentrations as a continuous variable with age as a covariate. Dogs were excluded from the genotyping population if they had suspected or confirmed causes of secondary HTG (e.g., diabetes mellitus, hypothyroidism, hyperadrenocorticism, or corticosteroid therapy).

A PCR-RFLP (restriction fragment length polymorphism) assay was designed to genotype the *APOE* variant. NEBcutter V2.0 (RRID SCR_010664) was used to identify differences in restriction enzyme sites for commercially available enzymes [31]. One enzyme, PsiI, was found to cut the reference sequence but not the sequence containing the *APOE* variant. Primer3 (RRID SCR_003139) was used to design primers to amplify a 382-base-pair (bp) product encompassing the *APOE* variant: forward primer 5′-AGATGTCACCTCCCTTCGTG-3′ and reverse primer 5′-CTGCGTGCATCCTCTTCC-3′ [32]. Standard polymerase chain reaction (PCR) amplification was performed using Qiagen HotStar Taq (Qiagen Sciences, Germantown, MD, USA) and 10 ng of DNA with 35 cycles, a 60 °C annealing temperature for 30 s, and a 72 °C elongation temperature for 30 s on an MJ Research PTC-100 thermal cycler (MJ Research, Waltham, MA, USA). The PCR product was incubated overnight at 37 °C with 1 unit of the PsiI enzyme. The PCR-RFLP assay products were resolved using gel electrophoresis. Dogs homozygous for the 3 bp *APOE* TATA box deletion had a single 379 bp product, dogs homozygous for the reference had a 181 bp product and a 201 bp product, and dogs heterozygous for the variant had all three products (181, 201, and 379 bp). Samples from one dog of each genotype were used as controls for the genotyping assay.

Genotyping for the *GPIHBP1* variant was performed using custom TaqMan SNP Genotyping Real-Time PCR Assay AN49E7V and TaqMan Genotyping Master Mix 4371353 (Applied Biosystems, Thermo Fisher Scientific Inc., Waltham, MA, USA). The TaqMan assays were performed using a real-time PCR instrument (CFX96 Touch, BIO-RAD, Hercules, CA, USA). The PCR conditions consisted of an initial denaturation at 95 °C for 10 min, followed by 40 cycles of denaturation at 95 °C for 15 s, and annealing/extension at 60 °C for 1 min. To ensure accuracy and reproducibility, all samples were run in duplicate. Results were analyzed using CFX Maestro 1.0 software (version 4.0.2325.0418, BIO-RAD, Hercules, CA, USA).

R statistical software was used for all statistical analyses (R, version 4.1.2, www.r-project.org (accessed on 18 November 2022); RRID SCR_001905) [33]. Data normality was evaluated using the Shapiro–Wilk test and quantile–quantile plot graphs (QQ plots). A multivariable regression model was fit on the genotyping cohorts with log-transformed triglyceride concentration as the dependent variable. The genotype for each variant was included (coded additively), along with male versus female and age (years) as covariates.

## 3. Results

### 3.1. Miniature Schnauzer WGS Cohort

The median fasting serum triglyceride concentration for the eight primary HTG dogs was 773 mg/dL (range 380–2089 mg/dL). The median age was 11 years (range 6–13 years). There were three females and five males; all were spayed and neutered, respectively. The median fasting serum cholesterol concentration was 314 mg/dL (range 162–491 mg/dL), with three dogs having concentrations above the upper limit of the reference interval for their respective laboratory. Three dogs had glomerular lipid thromboemboli diagnosed by evaluation of renal biopsy specimens by the International Veterinary Renal Pathology Service [9]; none were azotemic or hypoalbuminemic, but all were proteinuric with urine protein-to-creatinine ratios (UPCs) of 3.0, 6.0, and 9.3. Two additional HTG dogs were also proteinuric with UPCs of 2 and 2.2; neither were azotemic or hypoalbuminemic, but renal biopsies were not performed. The other HTG dogs did not have UPCs measured.

### 3.2. Candidate Gene Analysis

Three variants were identified in the six HTG candidate genes that passed all filtering criteria. These included an upstream promoter region variant in *APOE*, an intronic variant in *LMF1*, and a missense variant in *GPIHBP1*. Variant details, including genotype and allele frequencies, are provided in Table 2. Exonic variants that were excluded because of an allele frequency >0.10 were also extracted and are listed in Table S2.

The *APOE* variant was a three-base-pair deletion in the 5′ untranslated region. The *APOE* deletion resulted in a loss of the “TAT” of the TATA box, an important promoter sequence previously described in mouse and human *APOE* [34,35]. The base-wise conservation score for one of these three nucleotides indicated significant conservation (Table 2). The three deleted nucleotides were conserved in 57/67 vertebrate species (Table S3); the 10 species in which the deleted nucleotides were not conserved included 3 bird, 3 reptile, 3 mammal, and 1 amphibian species. The *APOE* promoter deletion was found in a heterozygous state in two of the eight whole-genome-sequenced Miniature Schnauzers with primary HTG, both of which were proteinuric with biopsy-confirmed glomerular lipid thromboemboli. The *APOE* variant was absent from the 613 non-Miniature Schnauzer dogs in the WGS database. In the 30 Miniature Schnauzers with unknown phenotypes from the WGS database, the variant allele frequency was 0.30.

The *LMF1* variant was an intronic single nucleotide polymorphism (SNP) located 456 bp upstream of exon 4. The nucleotide and surrounding 500 bp could not be identified in the human assembly GRCh38/hg38 (GenBank assembly GCA_000001405.15). Thus, a PhyloP conservation score could not be obtained.

The *GPIHBP1* missense variant (p.S91L, UniProt A0A8C0P969) was present in the Ly-6 protein domain, as determined by InterProScan (IPR016054, amino acids 87-162). The variant was predicted to have a neutral impact on the protein by SNPs&GO, PolyPhen2-HumDiv, and PolyPhen2-HumVar (0.14, 0.28, and 0.08, respectively) and was not conserved across vertebrate species. It was found in a heterozygous state in two of the eight whole-genome-sequenced Miniature Schnauzers with primary HTG; one had proteinuria, but neither had renal biopsies performed. It was also present in a heterozygous state in one non-Miniature Schnauzer in the WGS database (a Dachshund), but it was not present in any of the 30 Miniature Schnauzers with unknown phenotypes.

### 3.3. Variant Follow-Up Genotyping

One hundred and eight samples were available from Miniature Schnauzers with fasting triglyceride concentrations, including the 8 dogs with primary HTG used for WGS. These dogs were genotyped for the *APOE* promoter deletion (g.111237170_111237172del) and *GPIHBP1* missense variant (g.37746233C>T; p.S91L). The median fasting serum triglyceride concentration for the genotyping cohort was 180 mg/dL (range 14–2821 mg/dL). There were 68 dogs classified as primary HTG cases (TG concentration >108 mg/dL), including 41 males and 27 females, with a median age of 10.1 years (range 2.4–14.9 years). There were 40 dogs classified as controls (≤108 mg/dL), including 25 males and 15 females, with a median age of 9.0 years (range 6.0–13.1 years).

**Figure 1:**
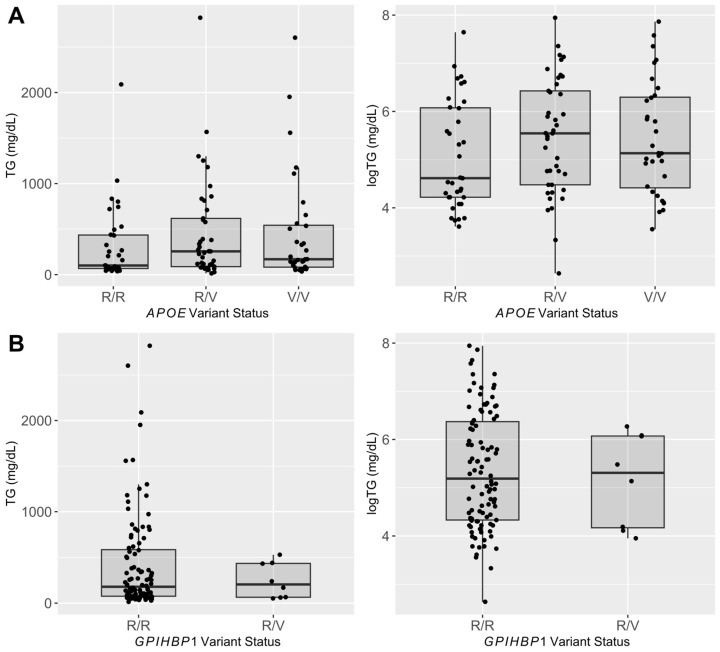
Range of triglyceride and log-transformed triglyceride concentration for the (**A**) *APOE* and (**B**) *GPIHBP1* genotypes. R, reference allele; V, variant allele.

Across the entire genotyping population, the *APOE* variant was present at an allele frequency of 0.49; 32 dogs were homozygous for the variant, 41 dogs were heterozygous, and 35 dogs were homozygous for the reference allele. The *GPIHBP1* variant was present in the genotyping population at an allele frequency of 0.04; none of the dogs were homozygous for the variant, 8 dogs were heterozygous, and 100 dogs were homozygous for the reference allele. The results of the multivariable regression model are shown in Table 3; there was no statistically significant effect of either the *APOE* or *GPIHBP1* variant genotype on log-transformed triglyceride concentration. In the model, age was a predictor of triglyceride concentration, but sex was not. The distribution of triglyceride concentrations by *APOE* and *GPIHBP1* variant genotypes is shown in Figure 1.

## 4. Discussion

In this study, we used WGS variant call data to evaluate for putative risk variants in six HTG candidate genes: *LPL*, *APOC2*, *APOA5*, *GPIHBP1*, *LMF1*, and *APOE*. Three variants met the filtering criteria: a 5′UTR deletion in *APOE*, an intronic *LMF1* SNP, and a missense variant in *GPIHBP1*. The *APOE* deletion was unique to the Miniature Schnauzer breed, involved a nucleotide that was conserved in vertebrate species, and altered an important promoter sequence. The *LMF1* SNP did not appear to be conserved, as the intronic region could not be mapped to the human reference genome. The *GPIHBP1* missense variant altered a poorly conserved base and was predicted to have a neutral impact on the protein. No association between the *APOE* or *GPIHBP1* variant genotypes and triglyceride concentration was identified in a cohort of 108 Miniature Schnauzers with known HTG phenotypes. The results of this study do not support a monogenic cause for HTG in Miniature Schnauzers in the six candidate genes evaluated.

Apolipoprotein E contributes to the metabolism of triglyceride-rich lipoproteins (very-low-density lipoproteins and chylomicrons) by controlling receptor-mediated clearance and acting as a cofactor for lipoprotein lipase [10]. The *APOE* promoter variant detected in this study is a deletion of the “TAT” in the TATA box. The TATA box is a sequence in the core promoter region and is involved in the initiation of transcription in TATA-containing genes [17]. Genes containing TATA box promoter motifs are enriched in metabolism pathways, such as carbohydrate, amino acid, and lipid metabolism [36]. Variants in the TATA box generally regulate gene expression but can lead to a range of effects, including alterations of the transcriptional start site or splicing defects [37]. Previously, a variant in the TATA box of apolipoprotein A-1 (*APOA1*) was associated with APOA1 deficiency and a resultant low concentration of high-density lipoproteins [38]. It is possible that the *APOE* TATA box deletion impacts the metabolism of triglyceride-rich lipoproteins through one of these mechanisms. However, the variant was not associated with triglyceride concentration in follow-up genotyping of a large Miniature Schnauzer cohort. Thus, it is unlikely that the *APOE* TATA box deletion is solely responsible for primary HTG in Miniature Schnauzers. The apparent exclusivity of the variant to the Miniature Schnauzer breed is of interest. It is possible that the *APOE* variant is a modifier or contributor to the condition instead of a monogenic cause. Previous studies have reported differences in lipid profiles and disease risk that are dependent on an individual’s *APOE* genotype [39,40,41,42,43]. Further experiments to measure *APOE* transcript and/or protein levels in Miniature Schnauzers might reveal an impact of the TATA box deletion on *APOE* transcription.

Lipase maturation factor 1 ensures the proper folding and assembly of LPL in the endoplasmic reticulum [19]. The *LMF1* variant is an intronic SNP that was located nearly 500 bp from the nearest exon. While deep intronic variants can be regulatory [44], the region containing the SNP did not map to the human reference genome. This suggests that the SNP does not reside within a conserved regulatory element and is unlikely to be a pathogenic variant. Analysis of *LMF1* cDNA sequence and expression would be ideal to more thoroughly investigate this variant.

Glycosylphosphatidylinositol-anchored HDL-binding protein 1 promotes the processing of triglyceride-rich lipoproteins and aids in the transport of lipoprotein lipase to the capillary lumen [23]. The *GPIHBP1* variant, a missense variant in the Ly-6 protein domain, alters a poorly conserved amino acid, and it was predicted to have a neutral impact on the protein by two pathogenicity prediction methods. The *GPIHBP1* variant was not associated with triglyceride concentration in a relatively large Miniature Schnauzer cohort. Additionally, the variant was rare in the Miniature Schnauzer cohort, and it was also detected in a non-Schnauzer breed. Thus, it is unlikely that the *GPIHBP1* variant is a major contributor to primary HTG in Miniature Schnauzers.

There are several possible explanations for why a monogenic cause for HTG was not discovered in these candidate genes. First, our analysis did not include an evaluation of structural variations (e.g., copy number variations, inversions, and translocations). Copy number variations have been identified in *LPL* and *GPIHBP1* in individuals with primary HTG and might contribute to disease severity [45,46,47]. A second possibility is that our filtering criteria for the allele frequency in non-Miniature Schnauzer breeds was too strict. There were additional variants identified (Table S2) that were excluded due to having allele frequencies greater than 0.10 in the non-Miniature Schnauzer breeds. If these variants have incomplete penetrance, their presence at a higher allele frequency in the non-Miniature Schnauzer breeds does not rule out a contribution to HTG risk, and association testing in a larger cohort would be required. A third possibility is that a putative causal variant for primary HTG in Miniature Schnauzers resides in a gene not included in this study. In humans, variants in the examined HTG candidate genes explain 42–54% of severe primary HTG cases [5,48]. Thus, in humans, half of primary HTG cases are due to other susceptibility genes. Other genes for consideration include several of the angiopoietin-like proteins (*ANGPTL3*, *ANGPTL4*, and *ANGPTL8*) that inhibit LPL activity; apolipoprotein C-III (*APOC3*), which inhibits LPL activity by displacing LPL from triglyceride-rich lipoproteins; lipase C, hepatic type (*LIPC*), which aids in the conversion of very-low-density lipoproteins and intermediate-density lipoproteins to low-density lipoproteins; and apolipoprotein A-IV (*APOA4*), which plays a role in triglyceride-rich lipoprotein secretion and metabolism [49,50,51]. Another, and perhaps the most likely, possibility is that primary HTG in Miniature Schnauzers is a polygenic or complex trait. In humans, HTG often develops from the cumulative effects of common and rare variations in multiple genes under the influence of the environment [48,52,53]. In dogs, triglyceride concentrations are influenced by age and diet, suggesting an environmental contribution to the disease [18,54,55,56].

## 5. Conclusions

In conclusion, a monogenic cause for primary HTG in Miniature Schnauzers was not identified in the six candidate genes evaluated in this study. Although three variants passed the filtering criteria, none had sufficient evidence to support a strong impact on primary HTG in Miniature Schnauzers. However, the apparent exclusivity of the *APOE* TATA box deletion in Miniature Schnauzers is of interest, and the variant might be a contributor to HTG manifestation. Given these findings and growing data on hyperlipidemia subtypes in Miniature Schnauzers, it is possible that HTG is a polygenic or complex trait in this breed.

## Figures and Tables

**Table 1:** Six candidate genes previously associated with primary hypertriglyceridemia in people.

Gene Symbol	Gene Name	Function
*LPL*	Lipoprotein lipase	Hydrolyzes circulating triglycerides [19]
*APOC2*	Apolipoprotein C-II	Essential cofactor for LPL activity [19,20]
*APOA5*	Apolipoprotein A-V	Stimulates LPL-mediated triglyceride hydrolysis [19,21]
*LMF1*	Lipase maturation factor 1	Essential for proper LPL function [19,22]
*GPIHBP1*	Glycosylphosphatidylinositol-anchored HDL binding protein 1	Facilitates LPL toward cell surface and promotes processing of triglyceride-rich lipoproteins [19,23]
*APOE*	Apolipoprotein E	Regulates clearance of chylomicron remnants and VLDL [10]

LPL, lipoprotein lipase; VLDL, very-low-density lipoprotein.

**Table 2:** Variants of interest identified by whole genome sequencing in Miniature Schnauzers with hypertriglyceridemia. Genomic locations are based on the UU-Cfam_GSD_1.0/canFam4 assembly (GenBank accession GCA_011100685.1).

Gene	Description	Variant Type	PhyloP Score	MS HTG				Other MS *				Non-MS Breeds ^†^			
				R/R	R/V	V/V	AF	R/R	R/V	V/V	AF	R/R	R/V	V/V	AF
*APOE*	g.111237170_ 111237172del	5′ UTR	1.95, 0.83, 0.43	6	2	0	0.13	14	14	2	0.30	613	0	0	0
*LMF1*	g.4020228C>A	intronic	NA ^††^	1	4	3	0.63	8	11	11	0.55	610	3	0	0.002
*GPIHBP1*	g.37746233C>T	missense	−3.66	6	2	0	0.13	30	0	0	0	599	1	0	0

AF, allele frequency; HTG, hypertriglyceridemia; MS, Miniature Schnauzers; R, reference nucleotide; V, variant. * Variant calls from 30 Miniature Schnauzers with unknown HTG phenotypes in the WGS database. ^†^ Three dogs in the non-MS breed population did not have a genotype call for the *GPIHBP1* variant. ^††^ A PhyloP score could not be determined for this nucleotide because the position could not be found in the human assembly GRCh38/hg38 (GenBank assembly GCA_000001405.15).

**Table 3:** A multivariable regression model for the effects of age, sex, *APOE* promoter deletion, and *GPIHBP1* missense variant genotype on log-transformed TG concentration.

Variable	Estimate of the Coefficient	Std. Error	*p* Value
*APOE* Genotype	0.18	0.14	0.20
*GPIHBP1* Genotype	−0.26	0.42	0.54
Age (years)	0.15	0.05	**0.006**
Sex (male)	0.01	0.23	0.97

*p*-Values in bold denote significance (<0.05).

## Data Availability

The whole genome data used in this manuscript are available in NCBI’s Short Read Archive under BioProject PRJNA937381 with the accession numbers: SRR25758627, SRR25758633, SRR25758630, SRR25758629, SRR25758631, SRR25758628, and SRR25758632.

## Supplementary Materials

The following supporting information can be downloaded at: https://www.mdpi.com/article/10.3390/genes15020193/s1, Table S1: Breed counts from the private WGS database; Table S2: Variants identified in targeted regions of six candidate genes. Table S3: The *APOE* TATA box sequence in 67 vertebrate species.
