# Supplementary material for: Sequence Analysis of Six Candidate Genes in Miniature Schnauzers with Primary Hypertriglyceridemia
Source: Genes (Basel). 2024 Jan 31;15(2):193. doi: 10.3390/genes15020193 (PMC10888295; doi:10.3390/genes15020193)
Supplement: Supplementary file 1 [file genes-15-00193-s001.zip › TableS1_12202023.pdf]

Table S1. Breed counts from the private WGS database.

Breed	#
Akita	1
American Staffordshire Terrier	2
American Foxhound	1
Australian Cattle Dog	2
Australian Shepherd	3
Bichon Frise	3
Border Collie	8
Border Terrier	1
Boston Terrier	5
Bouvier des Flandres	10
Boxer	37
Boykin Spaniel	3
Brittany Spaniel	3
Bulldog	28
Bullmastiff	19
Cairn Terrier	7
Cavalier King Charles Spaniels	23
Collie	5
Coonhound	1
Corgi	8
Dachshund	15
Doberman Pinscher	8
English Bulldog	6
English Cocker Spaniel	1
English Mastiff	1
French Bulldog	21
German Shepherd	27
Golden Retriever	46
Goldendoodle	2
Great Dane	25
Great Pyrenees	3
Havanese	2

Irish Setter	3
Irish Wolfhound	20
Labradoodle	2
Labrador Retriever	22
Lhasa Apso	3
Miniature Poodle	10
Miniature Schnauzer	30
Mixed Breed	9
Newfoundland	15
Pomeranian	13
Portuguese Water Dog	11
Pug	4
Rhodesian Ridgeback	4
Rottweiler	17
Scottish Deerhound	10
Scottish Terrier	6
Sheltie	9
Shih Tzu	1
Shiloh Shepherd	9
Siberian Huskey	16
Spanish Greyhound	1
Spinoni Italiano	1
Standard Poodle	28
Toy Poodle	4
Wachtelhund	2
Welsh Springer Spaniel	4
Welsh Terrier	1
Whippet	15
West Highland White Terrier	4
Yorkshire Terrier	42
**Total non-Miniature Schnauzer dogs**	**613**
**Total all dogs**	**643**
