# Supplementary material for: Sequence Analysis of Six Candidate Genes in Miniature Schnauzers with Primary Hypertriglyceridemia
Source: Genes (Basel). 2024 Jan 31;15(2):193. doi: 10.3390/genes15020193 (PMC10888295; doi:10.3390/genes15020193)
Supplement: Supplementary file 1 [file genes-15-00193-s001.zip › TableS3_12202023.pdf]

Table S3. The *APOE* TATA box sequence in 67 vertebrate species. The 3 bp that are deleted in the variant detected are shown in red to highlight their conservation across species.

Dog	TATA
Human	TATA
Chimp	TATA
Gorilla	TATA
Gibbon	TATA
Rhesus	TATA
Crab-eating macaque	TATA
Baboon	TATA
Green monkey	TATA
Marmoset	TATA
Squirrel monkey	TATA
Bushbaby	TATA
Chinese tree shrew	TATA
Squirrel	TATA
Less Egyptian Jerboa	TATA
Prairie Vole	TATA
Chinese Hamster	TATA
Golden Hamster	TATA
Mouse	TATA
Rat	TATA
Naked Mole-rat	TATA
Guinea Pig	TATA
Chinchilla	TATA
Rabbit	TATA
Pika	TATA
Pig	TATA
Alpaca	TATA
Bactrian Camel	TATA
Dolphin	TATA
Killer Whale	TATA
Tibetan Antelope	TATA
Cow	TATA
Sheep	TATA
Domestic Goat	TATA
Cat	TATA
Ferret	TATA
Panda	TATA
Pacific Walrus	TATA
Weddell Seal	TATA
Black Flying-fox	TATA
Megabat	TATA
Big Brown Bat	TATA

David's Myotis Bat	TATA
Little Brown Bat	TATA
Hedgehog	TATA
Shrew	TATA
Star-nosed Mole	TATA
Elephant	TATA
Cape Elephant Shrew	TATA
Manatee	TATA
Cape Golden Mole	TATA
Tenrec	TATA
Aardvark	TATA
Armadillo	TATA
Tasmanian Devil	TATA
Wallaby	TATA
Brush-tailed rat	TATA
Zebra Finch	CAGA
Green Sea Turtle	CAGG
Chinese Softshell Turtle	CAGG
Scarlet Macaw	GAG-
Opossum	GGGA
American Alligator	GGGT
X. tropicalis	TCTA
Collared Flycatcher	TGGG
Horse	TTTA
